# Photophobia and migraine outcome during treatment with galcanezumab

**DOI:** 10.3389/fneur.2022.1088036

**Published:** 2023-01-18

**Authors:** Francesca Schiano di Cola, Giulia Ceccardi, Marco Bolchini, Salvatore Caratozzolo, Paolo Liberini, Alessandro Padovani, Renata Rao

**Affiliations:** Neurology Unit, Department of Clinical and Experimental Sciences, ASST Spedali Civili, University of Brescia, Brescia, Italy

**Keywords:** migraine, CGRP, photophobia, galcanezumab, light, disability

## Abstract

**Background:**

Calcitonin gene-related peptide (CGRP) plays a pivotal role in migraine physiology, not only regarding migraine pain but also associated symptoms such as photophobia. The aim of the present study was to assess monoclonal antibodies targeting CGRP efficacy not only in terms of headache and migraine frequency and disability but also in reducing ictal photophobia.

**Material and methods:**

This is a retrospective observational study, conducted at the Headache Center–ASST Spedali Civili Brescia. All patients in monthly treatment with galcanezumab with at least a 6-month follow-up in September 2022 with reported severe photophobia during migraine attacks were included. Data regarding headache frequency, analgesics consumption, and migraine disability were collected quarterly. Moreover, patients were asked the following information regarding photophobia: (1) whether they noticed an improvement in photophobia during migraine attacks since galcanezumab introduction; (2) the degree of photophobia improvement (low, moderate, and high); and (3) timing photophobia improvement.

**Results:**

Forty-seven patients were enrolled in the present study as they met the inclusion criteria. Seventeen patients had a diagnosis of high-frequency episodic migraine and 30 of chronic migraine. From baseline to T3 and T6, a significant improvement in terms of headache days (19.2 ± 7.6 vs. 8.6 ± 6.8 vs. 7.7 ± 5.7; *p* < 0.0001), migraine days (10.4 ± 6.7 vs. 2.9 ± 4.3 vs. 3.6 ± 2.8; *p* < 0.0001), analgesics consumption (25.1 ± 28.2 vs. 7.6 ± 7.5 vs. 7.6 ± 8.1; *p* < 0.0001), MIDAS score (82.1 ± 48.4 vs. 21.6 ± 17.6 vs. 18.1 ± 20.5; *p* < 0.0001), and HIT-6 score (66.2 ± 6.2 vs. 57.2 ± 8.6 vs. 56.6 ± 7.6; *p* < 0.0001) was found. Thirty-two patients (68.1%) reported a significant improvement in ictal photophobia, with over half of the patients reporting it within the first month of treatment. Photophobia improvement was more frequent in patients with episodic migraine (*p* = 0.02) and triptans responders (*p* = 0.03).

**Conclusions:**

The present study confirms previous reports regarding galcanezumab efficacy beyond migraine frequency. In particular, over 60% of patients, in our cohort, documented a significant improvement also in reducing ictal photophobia. This improvement was, in most patients, moderate to high, and within the first 6 months of treatment, regardless of the clinical response on migraine frequency.

## 1. Introduction

Photophobia occurs in a wide range of ophthalmic and neurological disorders, the commonest of which is migraine (1, 2). It is a diagnostic criterion of migraine based on the International Headache Society (IHS) and is defined as hypersensitivity to light, causing avoidance (3). Moreover, it has been reported as the most bothersome associated symptom of migraine (4).

The neuropeptide calcitonin gene-related peptide (CGRP) is now recognized as a key player in the pathogenesis of migraine (5–7). CGRP is found in neurons of both the central and the peripheral nervous systems (respectively, CNS and PNS), and its receptors are widespread throughout the body, where it has been implicated in diverse functions (8). In the CNS, the CGRP has been mainly linked to nociceptive signaling, whereas in the periphery, it is the most potent vasodilatory peptide and contributes to neurogenic inflammation. Both the central and the peripheral effects of CGRP action are consistent with migraine symptoms, including photophobia.

All monoclonal antibodies are effective in migraine prophylaxis, in randomized clinical trials as well as in real-life settings (9–17), in difficult-to-treat patients with chronic migraine (18–20), with or without medication overuse (19, 21, 22) and in patients with multiple comorbidities, especially psychiatric (23–25), and migraine with aura (26).

Galcanezumab is a humanized IgG4 monoclonal antibody that selectively binds to and neutralizes CGRP (ligand). Galcanezumab binds to CGRP with high affinity (KD = 31 pM) and high specificity (>10,000-fold vs. related peptides adrenomedullin, amylin, calcitonin, and intermedin). The recommended dose is 120-mg galcanezumab injected subcutaneously monthly, with a 240-mg loading dose as the initial dose, in order to achieve steady state within one month.

Having established the efficacy of anti CGRP monoclonal antibodies in terms of migraine frequency, pain intensity and disability, current research is focusing beyond these aspects of migraine, such as interictal disability, quality of life and migraine associated symptoms. Response predictors are also being investigated, with unilateral pain and triptan response being associated with a consistent response to galcanezumab (27).

The aim of the present study was to assess galcanezumab efficacy–a monoclonal antibody that targets the CGRP molecule–in reducing standard variables of outcome (headache/migraine days, pain intensity, analgesics consumption, and migraine-related disability) and ictal photophobia.

## 2. Materials and methods

### 2.1. Standard protocol approvals and patient consent

This study received approval from the ethical standards committee on human experimentation (local ethics committee of the ASST Spedali Civili Hospital, Brescia: NP 3949, approved August 10, 2020). Full written informed consent was required from all participants.

### 2.2. Study design and participants

The present work is an observational study conducted at the Headache Center–Neurology Clinic at the Spedali Civili Hospital of Brescia.

The study included all adult patients with a diagnosis of HFEM or CM in prophylactic treatment with galcanezumab with a 6-month follow-up in September 2022. Inclusion criteria were as follows: documented history of migraine for at least 12 months, headache diary compilation in the 3 months prior to study enrolment, ≥8 migraine days per month for at least 3 months, ≥3 previous prophylactic failures, reported moderate-to-severe photophobia during migraine attacks.

Clinical and demographical information (disease duration, migraine-associated symptoms, and severity, triptans response, migraine localization, and previous prophylactic treatments) were collected at baseline (T0). Patients were treated with galcanezumab subcutaneous injection with a first loading dose of 240 mg at T0 and then 120 mg monthly. Data regarding headache frequency (monthly headache and migraine days–respectively, MHDs and MMDs), analgesics consumption, attacks' pain intensity (using the Numerical Rating Scale, NRS), and migraine disability (MIDAS and HIT-6 scores) were collected following three (T3) and six (T6) months of treatment. Moreover, patients were asked the following information regarding photophobia: (1) whether they noticed an improvement in photophobia during migraine attacks since galcanezumab introduction; (2) the degree of photophobia improvement (low, moderate, and high); and (3) since when they noticed the photophobia improvement.

### 2.3. Outcome measures

The objective of this analysis was to assess the clinical outcome of migraine patients in prophylaxis with galcanezumab, in terms of migraine frequency, disability, and associated symptoms, in particular, photophobia.

The primary endpoint was to assess MHDs, MMDs, pain intensity, analgesics consumption, and migraine disability (MIDAS and HIT-6 scores) at T0, T3, and T6.

The secondary endpoint was to evaluate, in patients reporting at T0 photophobia as a severe migraine-associated symptom, photophobia at T3 and T6. In particular, patients were asked whether they noticed improvement, worsening, or stability of ictal photophobia during treatment with galcanezumab. In case of improvement, patients were asked about the degree and timing of improvement.

The following secondary endpoints were also evaluated: (1) the correlation between migraine and photophobia outcome during treatment with galcanezumab; and (2) the correlation between patients' clinical characteristics and photophobia.

### 2.4. Statistical analysis

Shapiro–Wilk test and Levene test were used to assess the normality of the distribution and the homogeneity of variance. Continuous variables were described as mean and standard deviation or median and interquartile range as appropriate, and categorical variables were expressed as frequencies and percentages.

A one-way repeated measures ANOVA was conducted to test whether there were statistically significant differences in MMDs/MHDs, pain intensity, analgesics consumption, and migraine disability (MIDAS and HIT-6 scores) from baseline to T3 and T6. Bonferroni correction for multiple comparisons was applied.

Spearman correlation coefficient and Chi-square were conducted in order to assess the secondary endpoints.

Statistical significance was set at *p* < 0.05. Data analyses were carried out with SPSS software (version 22.0; Armonk, NY).

## 3. Results

Eighty patients were in treatment with galcanezumab in September 2022. Fifteen patients were excluded as their follow-up was < 6 months, whereas 18 patients were excluded as they did not report photophobia as a significant migraine-associated symptom at baseline. Thus, 47 patients were enrolled in the present study as they met the inclusion criteria.

Based on the main statistics performed and the number of subjects enrolled, a *post-hoc* analysis was performed in order to assess the estimated power (G power calculator), which resulted to be equal to 0.97.

Seventeen patients (36.2%) had a diagnosis of high-frequency episodic migraine, and 30 (63.8%) patients had chronic migraine. The mean age at baseline was 46.5 (9.1) years. Disease duration was, on average, 31.1 (10.4) years. Allodynia was reported by 29 patients (61.7%). A significant response to triptans (pain-free in 2 h) was reported by 33 patients (70.2%). Unilateral pain with side consistency was reported by 26 patients (55.3%). The median previously failed migraine preventive treatments was 5 (3–5.75). Medication overuse (MO) was reported by 34 patients (72.3%). All clinical and demographical data are summarized in Table 1.

**Table 1:** Clinical and demographical characteristics of all patients.

	**All patients ** **(*n* = 47)**	**Photophobia responders ** **(*n* = 32)**	**Photophobia non-responders ** **(*n* = 15)**	***p***
Diagnosis (*n*; %) - HFEM - CM	17 (36.2%) 30 (63.8%)	15 (88.2%) 17 (56.7%)	2 (11.8%) 13 (43.3%)	0.02^♯^
Age (mean; SD)	46.5 (9.1)	47.6 (9.4)	44 (8.2)	NS^∧^
Female gender (*n*; %)	40 (85.1%)	27 (67.5%)	13 (32.5%)	NS^♯^
Disease duration, years (mean; SD)	31.1 (10.4)	32 (10.6)	29.2 (10.2)	NS^∧^
Previous prophylactic treatments (median; range)	5 (3–5.75)	4 (3–5)	5 (3–7)	NS^§^
Allodynia (*n*, %)	27 (61.7%)	19 (70.4%)	8 (29.6%)	NS^♯^
Triptans responders (*n*; %)	33 (70.2%)	26 (78.8%)	7 (21.1%)	0.03^♯^
Unilateral pain (*n*; %)	26 (55.3%)	20 (76.9%)	6 (23.1%)	NS^♯^
Medication overuse (*n*; %)	34 (72.3%)	23 (67.6%)	11 (32.4%)	NS^♯^
Baseline MHDs (mean; SD)	19.2 (7.6)	17.03 (7.6)	23.6 (6.7)	NS^∧^
Baseline MMDs (Mean; SD)	10.4 (6.7)	9.7 (6.4)	12.6 (7.2)	NS^∧^
Baseline analgesics consumption (mean; SD)	25.1 (28.2)	21.09 (19.3)	26 (36)	NS^∧^
Baseline MIDAS score (mean; SD)	82.1 (48.4)	68.5 (39.5)	113.8 (69.8)	0.008^∧^
Baseline HIT-6 score (mean; SD)	66.2 (6.2)	64.6 (5.3)	65.7 (5)	NS^∧^
Baseline pain intensity, NRS score (mean; SD)	7.3 (1.1)	7.5 (1.1)	7.7 (0.8)	NS^∧^

N, number; HFEM, high-frequency episodic migraine; CM, chronic migraine; SD, standard deviation; MHDs, monthly headache days; MMDs, monthly migraine days.

∧ , Independent samples t-test;

♯ , Chi-square test;

§ , Kruskal–Wallist test.

**Figure 1:**
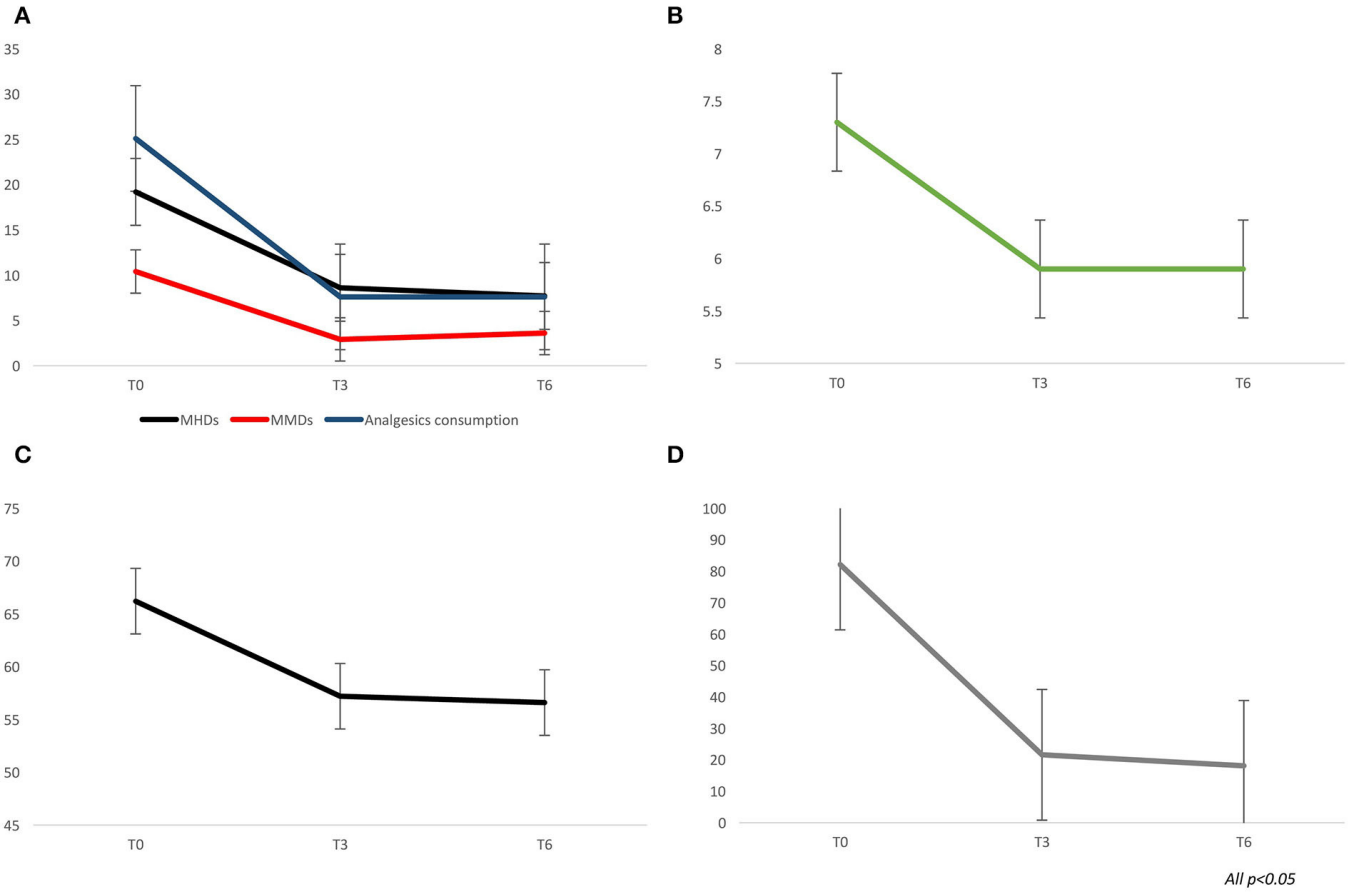
Migraine outcome during treatment with galcanezumab, at three (T3) and six (T6) months of treatment. **(A)** monthly headache and migraine days, analgesics consumption. **(B)** pain intensity–Numerical rating scale scores. **(C)** HIT-6 scores. **(D)** MIDAS scores.

**Figure 2:**
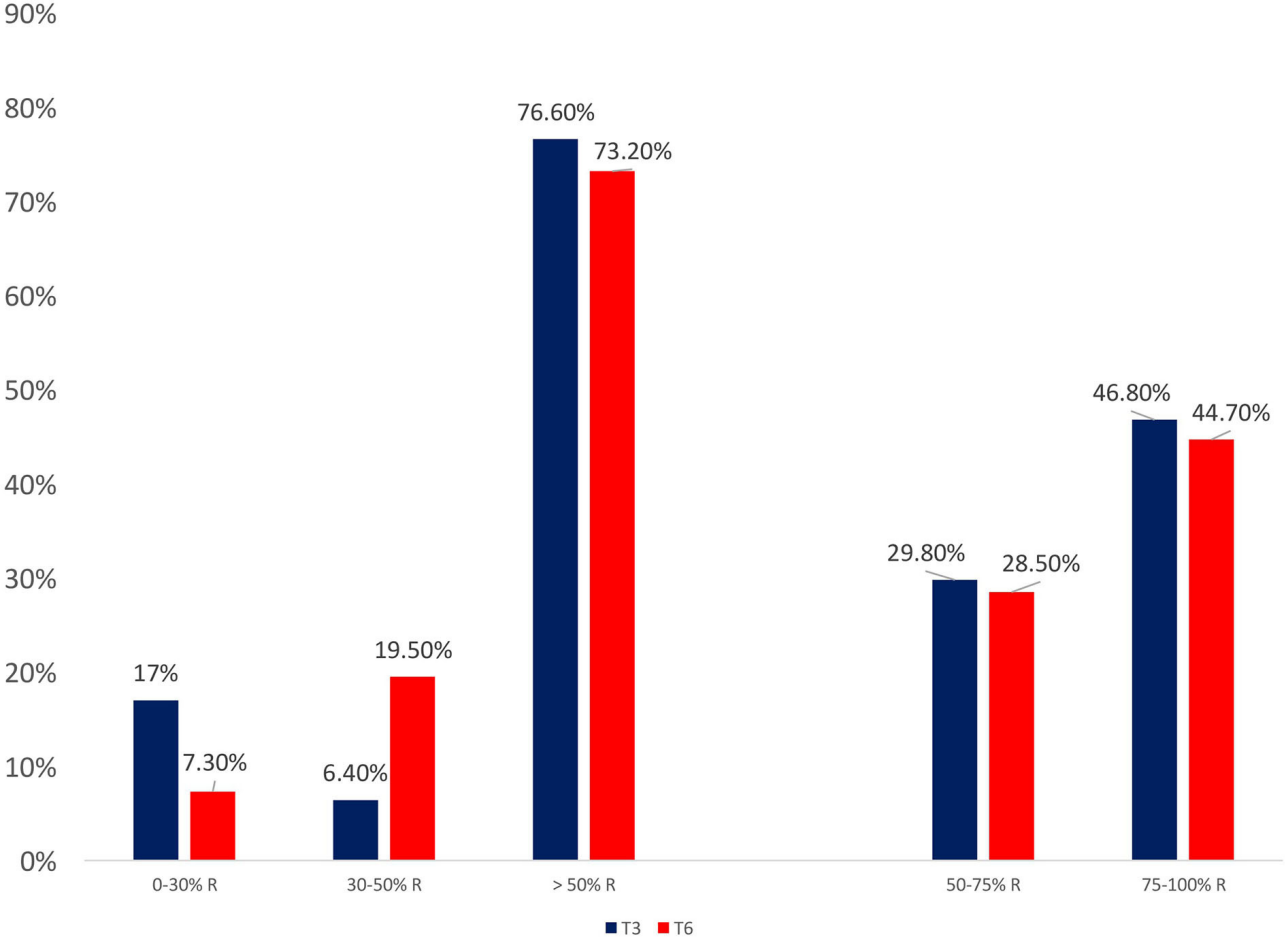
Response patterns during treatment with galcanezumab, at three (T3) and six (T6) months of treatment.

From baseline to T3 and T6, a significant improvement in terms of headache days (19.2 ± 7.6 vs. 8.6 ± 6.8 vs. 7.7 ± 5.7; *p* < 0.0001), migraine days (10.4 ± 6.7 vs. 2.9 ± 4.3 vs. 3.6 ± 2.8; *p* < 0.0001), analgesics consumption (25.1 ± 28.2 vs. 7.6 ± 7.5 vs. 7.6 ± 8.1; *p* < 0.0001), pain intensity–NRS score (7.3 ± 1.1 vs. 5.9 ± 1.6 vs. 5.9 ± 1.5; *p* < 0.0001), MIDAS score (82.1 ± 48.4 vs. 21.6 ± 17.6 vs. 18.1 ± 20.5; *p* < 0.0001), and HIT-6 score (66.2 ± 6.2 vs. 57.2 ± 8.6 vs. 56.6 ± 7.6; *p* < 0.0001) was found (see Figure 1). At T3 and T6, the percentage of responders (patients who lost >50% of their baseline headache/migraine days) was 76.6 and 73.2%, respectively (see Figure 2). The percentage of super responders (patients who lost >75% of their baseline headache/migraine days) at T3 and T6 was 46.8 and 45.7%, respectively.

Thirty-two patients (68.1%) reported a significant improvement of ictal photophobia, of which 6 patients (18.8%) reported a slight improvement, 11 (34.4%) a moderate improvement, and 15 (46.9%) a high improvement. The improvement in photophobia was observed within the first month of treatment in 18 patients (56.3%), with 12 patients (37.6%) reporting a significant improvement within the first 6 months of treatment. Two patients (6.2%) reported an improvement in photophobia only during the second treatment cycle (first and third months of retreatment).

Photophobia improvement, although more frequent in responders, did not document a statistically significant difference between responders and non-responders at T3 and T6 (see Table 2). Although not significant (*p* = 0.14), photophobia improvement was more frequent whose migraine had a unilateral constant localization compared to those without such characteristic (76.9 vs. 57.1%).

**Table 2:** Frequency of photophobia responders and non-responders according to the migraine responders rate.

	**Photophobia responders (*n* = 32)**	**Photophobia non-responders (*n* = 15)**	***p***
Responders rates T3 (*N*; %) - < 30% - 30–50% - >50%	5 (33.3%) 0 10 (66.7%)	3 (9.4%) 3 (9.4%) 26 (81.2%)	NS^♯^
Responders rates T6 (*N*; %) - < 30% - 30–50% ->50%	2 (14.3%) 4 (28.6%) 8 (57.1%)	1 (3.7%) 4 (14.8%) 22 (81.5%)	NS^♯^

N, number;

♯ , Chi-square test.

Moreover, photophobia improvement was more frequent in triptan responders compared to non-responders (69.6 vs. 30.4%; *p* = 0.03). Considering migraine diagnosis, 88.2% of patients with HFEM documented a photophobia improvement, compared to 55.7% of CM patients (*p* = 0.02).

Baseline migraine disability (MIDAS score) was higher in patients who did not document a photophobia improvement during treatment compared to those who did (113.8 ± 69.8 vs. 68.5 ± 39.5; *p* = 0.008). Lower migraine disability was also documented during treatment, at T3 and T6, in patients who did document an improvement in ictal photophobia compared to those who did not (T3: 16.7 ± 15.1 vs. 31.6 ± 24.3, *p* = 0.01; T6: 11.7 ± 11.5 vs. 29.3 ± 26.4, *p* = 0.04).

## 4. Discussion

The present study confirms previous data regarding galcanezumab efficacy in migraine prevention. Following 3 and 6 months of treatment, a significant improvement in migraine/headache days, pain intensity, and analgesics consumption was found. A similar improvement was also observed in terms of migraine disability (MIDAS and HIT6 scores).

Since photophobia has been reported as the most bothersome migraine-associated symptom, the aim of the present study was to evaluate whether ictal photophobia also improved during treatment with galcanezumab. In our cohort, up to 68% of patients reported photophobia improvement since galcanezumab introduction. Most of the patients who did notice such improvement reported it within the first month of treatment. Moreover, the improvement was reported as high (on a scale consisting of the three options: low, moderate, and high) by nearly half of the patients. Of notice, two patients reported ictal photophobia improvement only during their second treatment cycle. Interestingly, photophobia improvement was independent of migraine improvement. However, patients who documented a significant response to galcanezumab regarding ictal photophobia documented lower levels of migraine disability, both at baseline and during treatment. The only significant correlations with photophobia improvement were diagnosis and triptans response. In particular, a significant improvement in ictal photophobia was more frequent in those with a diagnosis of episodic migraine and those who displayed a significant response to triptans.

Individuals have different thresholds for light sensitivity, and it has been found that migraine patients tend to have lower thresholds compared to the general population (28), not only during headaches but also between attacks (29). A “light-pain matrix” (30) has been hypothesized bridging together retinal structures and various brain area found to be involved in the painful sensation of light processing reported as photophobia, such as thalamus, trigeminal nucleus, superior colliculus and the visual cortex (31). Recently, a class of retinal ganglion cells has been discovered and named intrinsically photosensitive retinal ganglion cell (IPRGC), also known as melanopsin cells (32). They respond to bright light and project their axons to the Edinger–Westphal and suprachiasmatic nuclei, involved in circadian rhythms and pupillary response to light, as well as thalamic nuclei (33–36). It has been hypothesized that these ganglions might be involved in the “light pain matrix” also by firing directly to the trigeminal nerve and trigeminal nerve efferents in turn causing ocular vasodilation and activation of pain-sensing neurons in blood vessels (37).

Neuropeptide CGRP has been found to be play a pivotal role in migraine pathogenesis (37–40). Moreover, various studies linked light aversive behavior in mice to CGRP, in both wild-type mice (41) and migraine models as hRAMP1 mice (42, 43). The effect of CGRP on photophobia seems to be in part mediated by its vasomotor activity but in part independent of it (42). The main site of action for CGRP, beyond the meninges, is the eye and trigeminal ganglia. With respect to the eye, bright light stimulus has previously been shown to evoke neural activity in central trigeminal neurons in rats, which could be inhibited by intravitreal injection of phenylephrine (vasoconstriction) (44). The hypothesis is that CGRP-mediated photophobia might be secondary to vascular events within the eye. CGRP vascular receptors have, indeed, been found within the eye and intravitreal injections of CGRP have been found to affect intraocular pressure (45). In contrast, involvement of the trigeminal system has been suggested by reports that intraganglionic injection of CGRP induces both photic sensitivity and facial allodynia in rodents (46).

Nonetheless, CGRP must also have non-vasomotor activities because some light aversive behavior was still observed in mice also in the presence of vasoconstrictors (41). At the vasculature, CGRP actions on smooth muscle and endothelium can potentially release cytokines, with consequent nociceptors sensibilization (47). Another plausible site of action is the meninges, which are densely populated with immune cells, in particular mast cells. Mast cells have been implicated in the sensitization of sensory neurons in migraine, and CGRP receptors have been reported in mice (48). Also, lymphocytes T and dendritic cells express CGRP receptors (49), possibly being involved in trigeminal sensitization.

On these premises, it is not surprising that galcanezumab, a potent anti-CGRP ligand monoclonal antibody, was found to be effective also in reducing photophobia in a cohort of migraine patients, regardless of migraine improvement. Photophobia improvement was more frequent in episodic compared to chronic migraine patients, possibly related to a lower level of central and peripheral sensitization in the latter patients. Of notice, photophobia was significantly more frequent in triptan response. This finding is in line with previous reports of an association between triptan response and erenumab and onabotulinumtoxin A response (50–52). The common action on the trigeminovascular systems between triptans and erenumab/onabotulinumtoxin A (53) was hypothesized as the key factor. Moreover, it has been found that triptan responders document higher ictal CGRP levels compared to non-responders (54). In triptan non-responders, pain neurotransmitters different from CGRP might be important in the generation of migraine; thus, triptan non-responders might be less responsive to CGRP-targeted treatments (50).

We recognize two major limitations in the present study: (1) the small cohort; and (2) the qualitative assessment of photophobia improvement.

Future research will be needed to replicate our findings, perhaps on a larger cohort, and investigate predictors of response. Moreover, the efficacy of CGRP monoclonal antibodies and other migraine-associated symptoms, such as dopaminergic symptoms, nausea, phono-, and osmophobia should be investigated.

## Data availability statement

The data that support the findings of this study are available from the corresponding author upon reasonable request.

## Ethics statement

The studies involving human participants were reviewed and approved by NP3949–Local Ethics Committee of the ASST Spedali Civili Hospital, Brescia. The patients/participants provided their written informed consent to participate in this study.

## Author contributions

FS: study conception and design, acquisition, analysis and interpretation of data, and drafting of manuscript. GC: acquisition and interpretation of data. MB: acquisition of data. SC, PL, and AP: critical revision and drafting of manuscript. RR: study conception and design and critical revision. All authors contributed to the article and approved the submitted version.
